# A data assimilation framework to predict the response of glioma cells to radiation

**DOI:** 10.3934/mbe.2023015

**Published:** 2022-10-08

**Authors:** Junyan Liu, David A. Hormuth, Jianchen Yang, Thomas E. Yankeelov

**Affiliations:** 1 Department of Biomedical Engineering, The University of Texas at Austin, 107 W Dean Keeton Street Stop C0800, Austin, TX 78712, USA; 2 Department of Diagnostic Medicine, The University of Texas at Austin, 1601 Trinity St Bldg. B Stop Z0300, Austin, TX 78712, USA; 3 Department of Oncology, The University of Texas at Austin, 1601 Trinity St Bldg. B, Austin, TX 78712, USA; 4 Oden Institute for Computational Engineering and Sciences, The University of Texas at Austin, 201 E. 24^th^ Street POB 4.102 Stop C0200, Austin, TX 78712, USA; 5 Livestrong Cancer Institutes, The University of Texas at Austin, 1601 Trinity St. Bldg. B Mail Stop Z1100, TX 78712, USA; 6 Department of Imaging Physics, The University of Texas MD Anderson Cancer Center, PO Box 301402, Houston, TX, 77230-1402, USA

**Keywords:** mathematical model, mechanism-based, biology-based model, calibration, time-resolved microscopy, *in vitro*, brain cancer, glioblastoma

## Abstract

We incorporate a practical data assimilation methodology into our previously established experimental-computational framework to predict the heterogeneous response of glioma cells receiving fractionated radiation treatment. Replicates of 9L and C6 glioma cells grown in 96-well plates were irradiated with six different fractionation schemes and imaged *via* time-resolved microscopy to yield 360- and 286-time courses for the 9L and C6 lines, respectively. These data were used to calibrate a biology-based mathematical model and then make predictions within two different scenarios. For Scenario 1, 70% of the time courses are fit to the model and the resulting parameter values are averaged. These average values, along with the initial cell number, initialize the model to predict the temporal evolution for each test time course (10% of the data). In Scenario 2, the predictions for the test cases are made with model parameters initially assigned from the training data, but then updated with new measurements every 24 hours *via* four versions of a data assimilation framework. We then compare the predictions made from Scenario 1 and the best version of Scenario 2 to the experimentally measured microscopy measurements using the concordance correlation coefficient (CCC). Across all fractionation schemes, Scenario 1 achieved a CCC value (mean ± standard deviation) of 0.845 ± 0.185 and 0.726 ± 0.195 for the 9L and C6 cell lines, respectively. For the best data assimilation version from Scenario 2 (validated with the last 20% of the data), the CCC values significantly increased to 0.954 ± 0.056 (p = 0.002) and 0.901 ± 0.061 (p = 8.9e-5) for the 9L and C6 cell lines, respectively. Thus, we have developed a data assimilation approach that incorporates an experimental-computational system to accurately predict the *in vitro* response of glioma cells to fractionated radiation therapy.

## 1. Introduction

The post-surgical standard-of-care for malignant gliomas, including glioblastoma multiforme (GBM), is adjuvant radiation and chemotherapy with temozolomide [1]. Due to the diffuse nature of GBM, complete resection may not be tenable [2], and thus radiation is required. In spite of this aggressive therapy, the prognosis for patients battling GBM remains poor with a median survival of only 15 months [3]. This high failure rate is thought to be at least partially due to the heterogeneous response different patients have to standard radiotherapy plans [4]. It is widely believed that treatment plans designed for each patient’s unique circumstances may improve the overall response rate [5]. To achieve this degree of individualized therapy requires a mathematical model that can be initialized with patient-specific parameters to make accurate patient-specific predictions of response. Given that radiation plans most commonly consist of many small fractions given over an extended period, incorporating follow up measurements into the modeling framework is of great importance and may enable adaptive treatment planning.

Data assimilation is an established set of techniques by which newly available data are integrated with the predictions of a mathematical model to refine the characterization of a system that is dynamically changing in time (and/or space) [6]. While this mathematical technique is widely used in (for example) weather forecasting [7,8], it is not commonly employed for planning, predicting, or optimizing the treatment of cancer. In the specific context of radiation therapy, the lack of a widely-accepted mathematical model of radiation response that explicitly incorporates temporal dynamics has fundamentally limited the application of data assimilation in radiation therapy. More specifically, the mathematical model employed in the standard-of-care setting for planning radiation dose schedules is the linear-quadratic (LQ) model [9]. The LQ model is most typically employed to compute the “surviving fraction” of tumor cells after a specific radiation dose, rather than the temporal response of tumor cells to radiation. Thus, it is unclear how data assimilation could significantly improve the predictions of the LQ model.

In recent years there has been substantial advances in not only constructing mathematical models that capture the spatial and temporal growth and response of tumors to radiation [10,11], but also in populating these models with the appropriate quantitative data [12,13]. One approach proposed by Rockne *et al*. [14] leveraged both anatomical and functional imaging data to modulate the spatial efficacy of radiotherapy as a function of tissue oxygenation. Compared to the assumption of uniform radiation sensitivity, reduced error was observed in tumor predictions when the efficacy of radiotherapy was modulated as a function of tissue oxygenation. At the pre-clinical level, Hormuth *et al*. developed a family of biophysical models based on a reaction-diffusion diffusion equation coupled to tissue mechanical properties [15]. The model was applied in a murine model of glioma and was able to accurately predict the spatiotemporal evolution of tumor growth following radiation therapy. Progress has also been made in the pre-clinical setting employing time resolved microscopy which provides the opportunity to acquire highly temporally resolved data. For example, Liu *et al*. collected irradiated tumor cells response *in vitro* every four to six hours *via* microscopy [16]. These data were paired with a mathematical model explicitly incorporating the temporal effects of radiation on tumor cells to predict the temporal development of 9L and C6 glioma cells in response to radiation. Brüningk *et al*. also employed time-resolved microscopy to observe response of tumor spheroids to radiation and hyperthermia treatments [17]. The resulting response dynamics were modeled using a cellular automaton model which accounts for cellular damage and the ability to repair damage in response to multi-modality treatments. Common to all of the above studies is the use of quantitative imaging to non-invasively, and repeatedly, interrogate tumor status before, during, and after treatment [12] thereby providing an avenue to incorporate the methods of data assimilation to update and refine model predictions. Indeed, there have been some early efforts at linking mathematical modeling, imaging, and data assimilation to advance predictive modeling.

In an early simulation study, Kostelich *et al*. [18] investigated the feasibility of applying data assimilation to modeling the growth of a simulated glioblastoma. Their approach combined Kalman-filter based data assimilation with simulated MRI data to predict the growth of an *in silico* tumor for one year with six equally spaced updates to their model forecasts. Another approach was taken by Zahid *et al*. [19] who developed a model that featured a tumor carrying-capacity that was reduced when exposed to radiation therapy. The model is initialized with population data and updated with patient-specific information as it became available, resulting in accurate predictions of patient outcomes. We now seek to build on these efforts to develop a data assimilation approach that makes use of highly time-resolved data and a mathematical model which explicitly accounts for the dynamic response of cancer cells to radiation.

We have recently developed an experimental-computational framework that employs time-resolved microscopy data to predict the response of tumor cells to fractionated radiation [20]. To enable predictions prior to the start of treatment, this framework utilized the initial number of tumor cells, the planned radiation schedule, and model parameters obtained from a training data set. While the overall predictions were strong (concordance correlation coefficients above 0.75 for both cell lines and all fractionation schemes), the heterogeneity of response makes it challenging to accurately predict all replicates. That is, significant prediction error can arise for replicates with the same initial conditions and fractionation plans but with very different responses. We now propose to extend this formalism by employing a practical data assimilation strategy (similar to that of Zahid *et al*. [19]) to update those predictions on a tumor specific basis. To accomplish this task, the model parameters describing the key radiobiological processes are updated every 24 hours based on new microscopy measurements to allow for refining the model prediction. By linking a mathematical model that explicitly incorporates the dynamic effects of radiation therapy with quantitative imaging data through data assimilation, we can not only quantify the underlying radiobiology, but also achieve high predictive accuracy as verified by six different fractionation schemes on both 9L and C6 glioma cell lines.

## 2. Materials and methods

### 2.1. Cell culture and radiation treatment

As the details of cell culture and radiotherapy experiments have been described previously [20], here we provide only the salient information to describe our dataset. 9L and C6 cell lines were seeded at 1,000 to 10,000 cells per well on 96 well plates. We then irradiated the cell lines with a total dose of either 0 Gy (control), 16 Gy or 20 Gy at a fixed dose rate of 1.5 Gy/min. In the 16 Gy group, cells are irradiated with either four fractions of 4 Gy, three fractions of 5.3 Gy or two fractions of 8 Gy. In the 20 Gy group, cells are irradiated with either four fractions of 5 Gy, three fractions of 6.7 Gy or two fractions of 10 Gy. There is a 24-hour interval between every fraction, which mimics the classical treatment schedule used in clinical settings. (Please see Figure S1 in the supplementary material for the detailed treatment schedule.) Immediately after the first fraction, images are collected every four to six hours for up to 330 hours *via* the Incucyte S3 Live Cell imager (Essen Bioscience, Michigan). The images are then segmented to estimate live cell confluences (i.e., percentage of area covered by live cells) at each imaging time point *via* a histogram-based pipeline described in [16]. The result is a time course of living cell number at each imaging time point for each well. Overall treated replicates and radiation schemes, we obtained a total of 360 time courses of the 9L line, and 286 time courses for the C6 line.

### 2.2. Multi-compartment model of glioma cells to fractionated radiation therapy

We previously proposed and validated a biology-based, data-driven model. While the full derivation is described in [20], we now present the salient details. We quantify the growth of untreated tumor cells at time t, N(t), by a combination of the Allee effect [21] and logistic growth. Biologically, the Allee effect describes the cooperation between cells at low confluence *via* the constant A, while logistic growth describes the growth of the cells up to a carrying capacity, θ, determined by a combination of limited nutrient availability and physical space. The resulting equation is given by:

(1) $$\frac{dN(t)}{dt}=k_{p}\cdot N(t)\cdot \underset{Allee\;effect}{\underset{︸}{\left(\frac{N(t)}{\theta }+A\right)}}\cdot \underset{logistic\;growth}{\underset{︸}{\left(1-\frac{N(t)}{\theta }\right)}},$$

where $k_{p}$ is the proliferation rate. The parameters $k_{p}$, θ, and A, are obtained by calibrating Eq. (1) to the N(t) time courses obtained from untreated cells and then held fixed for the remainder of the analyses.

After exposure to radiation, we account for two death pathways that occur at different time scales. First, within hours post-radiation, the DNA repair pathways are engaged [22], but the unrepaired double-strand breaks (DSBs) may lead to acute apoptosis due to the activation of DNA-dependent protein kinase and p53 [23]. Secondly, clustered DSBs (i.e., DSB foci in close proximity) can cause DNA misrepair and chromosome aberrations [24]. Although misrepair does not kill cells instantaneously, it accumulates within the cells’ genomes within weeks following radiation [25] and eventually leads to mitotic catastrophe [26]. Additionally, there are multiple mechanisms [27], including cell cycle checkpoints [28], that contribute to driving a fraction of the irradiated cells into a senescent population. To account for these three pathways, we extend Eq. (1) to include early death, late death, and entrance into senescence:

(2) $$\frac{dN_{p}(t)}{dt}=(k_{p}-\underset{\text{late death}}{\underset{︸}{k_{ld}(t,D,N_{0})}})\cdot \underset{\text{Allee effect}}{\underset{︸}{\left(\frac{N_{p}(t)+N_{s}(t)}{\theta }+A\right)}}\cdot \underset{\text{logistic growth}}{\underset{︸}{N_{p}(t)\cdot \left(1-\frac{N_{p}+N_{s}(t)}{\theta }\right)}},\;\;-\underset{\text{early death}}{\underset{︸}{k_{ed}(t,D,N_{0})\cdot N_{p}(t)}}-\underset{\text{Conversion to senescence}}{\underset{︸}{k_{ps}(D_{total})\cdot N_{0}\cdot N_{p}(t)}}$$

where $N_{p}(t)$ and $N_{s}(t)$ are the proliferating and senescent cells, respectively, $k_{ed}$ and $k_{ld}$ are the early and late death rates, respectively, and $k_{ps}$ is the conversion rate from the proliferating to the senescent compartment. Observe that $k_{ld}$ and $k_{ed}$ are functions of time t, dose per fraction D, and seeding density $N_{0}$, while $k_{ps}$ is a function of the total dose, $D_{total}$. We next describe the specific formulations of $k_{ld}$, $k_{ed}$, $k_{ps}$ , and $N_{s}(t)$.

The early death term is formulated as in Eq. (3):

(3) $$k_{ed}(t,N_{0},D)=\sum_{i=1}^{\begin{array}{c} fraction\\ number \end{array}}k_{acute}(N_{0})\cdot \underset{\text{DSB repair}}{\underset{︸}{f_{DSB}(t,D)}},$$

where $f_{DSB}$ is the fraction of unrepaired DSBs and was estimated from the concentration of gamma-H2AX [16] *via* flow cytometry. $k_{acute}$ is the early death rate, and is hypothesized to be proportional to seeding density with constant $k_{acute,N}$ as in Eq. (4)

(4) $$k_{acute}(N_{0})=k_{acute,N}\cdot N_{0}.$$

The late death is formulated as Eq. (5):

(5) $$k_{ld}(t,D,N_{0})=\sum_{1}^{fractionNumber}k_{accum}(D,N_{0})\cdot t\cdot e^{-r.t},$$

where the death rate first increases as misrepair accumulates over time (thus multiply time t), and then decreases due to the decay of radiation efficacy with rate r. $k_{accum}$ is the late death rate, and is hypothesized to be proportional to both seeding density with constant $\alpha_{accum,N}$, and doses with constant $k_{accum,D}$ respectively as in Eq. (6):

(6) $$k_{accum}(D,N_{0})=(\alpha_{accum,N}\cdot N_{0}+1)\cdot k_{accum,D}\cdot D.$$

Finally, the senescent cell compartment $N_{s}(t)$ represents the conversion from the proliferating cell compartment, $N_{p}(t)$, as in Eq. (7):

(7) $$\frac{dN_{s}(t)}{dt}=\underset{\text{Conversion to senescence}}{\underset{︸}{k_{ps}(D_{total})\cdot N_{0}\cdot N_{p}(t)}}.$$

For simplicity, we treat $k_{ps}$ as a function of total dose; i.e., $k_{ps}(D_{total}=16\;\text{Gy})$ and $k_{ps}(D_{total}=20\;\text{Gy})$ are considered two separate parameters. (Please see ref. [20] for the complete derivation).

### 2.3. Training, test, and validation groups

The control group (0 Gy) is used to estimate $k_{p}$, θ, and A which are then fixed throughout the study. The irradiated 9L and C6 datasets are divided into three groups each: 70% for training (252 9L samples, 197 C6 samples), 10% for testing (36 9L samples, 25 C6 samples), and 20% for validation (72 9L samples, 64 C6 samples). The training group can be viewed as historical data, and this data is simultaneously fit to our model to estimate parameters for predicting the time courses of the validation and test groups. As two samples can have the same treatment schedule and seeding density, their response can still vary due to the differences in passage number, cell cycle distribution, phenotype and genotype. Thus, in the testing and validation groups, new data become available every 24 hours and are used to update the model predictions.

Please see supplementary material table S1 for a list of parameter definitions, and supplementary material table S2 for the estimated values of $k_{p}$, θ, and A for the 9L and C6 lines, respectively.

### 2.4. Numerical implementation

We implement Eqs. (2) - (7) *via* the finite difference method using explicit forward Euler formation with time step equals 0.01 hours in MATLAB R2019b (MathWorks, Natick, MA). The initial conditions of the model system are $N_{p}(0)$, the cell confluence measurement at time 0, and $N_{s}(0)=0$ (i.e., assuming no senescent cells right after radiation). Curve fitting is implemented *via* MATLAB function ‘lsqnonlin’, which solves the nonlinear least squares problems *via* the Levenberg-Marquardt algorithm. The standard deviation of the resulting estimated parameters is then computed *via* MATLAB function ‘nlparci’. All model calibrations and numerical methods were performed on a personal computer with a 3.7 GHz Intel i7-10900K with 10 cores and 32 GB memory. Using this system, a single calibration to the model took less than 2 minutes.

### 2.5. Prediction via population-based global parameters (Scenario 1)

**Figure 1.**
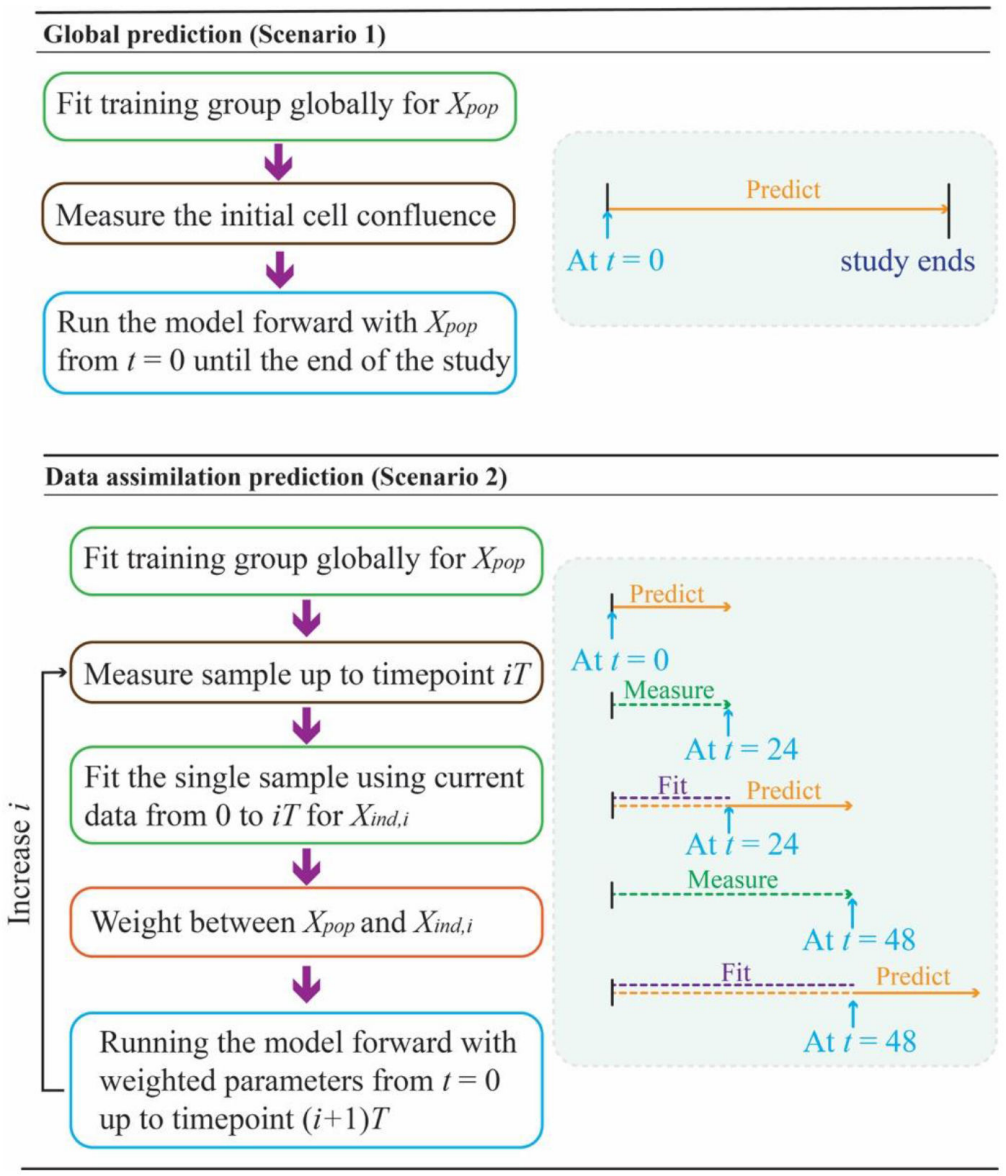
Model prediction framework. Two types of predictions are illustrated in the flow chart, where i is the data assimilation step and T is the time interval between predictions (which is fixed at T = 24 hrs in this study). The global prediction (top panel), where parameters are acquired from the training group ($X_{pop}$) and fixed throughout the time course. This prediction relies only on the initial condition of the test sample; i.e., if two samples have the same initial cell confluence, the model prediction will be the same. Conversely, the prediction of the data assimilation framework (bottom panel) depends on both the initial condition and previous measurements from the population and the sample itself. The model will keep updating the prediction as time advances as more information about the test sample is acquired to yield a new set of model parameters at time step $i\;(X_{ind,i})$.

After assigning $k_{p}$, θ, and A, we then simultaneously fit all training curves (regardless of radiation dose or seeding density) globally to Eqs. (2) - (7) to obtain a single set of estimates for the remaining model parameters $k_{acute,N}$, $\alpha_{accum,N}$, $k_{accum,D}$, r, and $k_{ps}$. The mean of the resulting parameters is denoted as $X_{pop}$ with deviation $\sigma_{pop}$. (For detailed numerical implementation and curve fitting, please see our previous work [20].) For a new sample from the (for example) testing group, we take its initial conditions (i.e., initial confluence and fractionated radiation scheme) and predict its temporal dynamics from time 0 to the end of the study *via* Eqs. (2) - (7) with the model parameters assigned as $X_{pop}$ as shown in the “global prediction” section (upper portion) of Figure 1. Note that the global prediction here only reflects the average treatment response based on the training population, and is not individualized beyond its initial conditions and radiation schedule. Thus, this global approach will yield identical predictions for two samples with the same seeding density and radiation dose when running the model forward with $X_{pop}$.

### 2.6. Prediction via data assimilation (Scenario 2)

#### 2.6.1. Basic Framework

Data assimilation is performed on the testing and validation groups as outlined in the bottom portion of Figure 1. First suppose we have already obtained time-resolved microscopy data from a new sample from 0 to iT hours with corresponding cell confluences N(t=0) to N(t=iT), where i is a positive integer and T is the time interval over which we want to make a prediction (T=24 hours in this study). The goal is to now make predictions from time iT to (i+1)T, which means the model parameters and the corresponding prediction are refined every 24 hours. Compared to Scenario 1, Scenario 2 updates the prediction by incorporating the information from new observations, and thus can better characterize the heterogeneity between samples.

#### 2.6.2. Sample-Specific individual parameters

We first fit all known measurements of this new sample (i.e., cell confluence from N(0) to N(iT)), to Eqs. (2), (3), (5), and (7) to obtain individual parameters $k_{acute}$, $k_{accum}$, r, and $k_{ps}$, denoted as $X_{ind,i}$, the individual parameters for step “i”. The cost function for this curve fit is given by Eq. (8):

(8) $$cost_{basic}=\sum_{j=1}^{\begin{array}{c} curve\\ number \end{array}}\sum_{t=0}^{iT}{\left(N(t)-model(X_{ind,i},t)\right)}^{2}.$$

Note that Eqs. (4) and (6) are not used when fitting an individual sample; i.e., we fit for $k_{acute}$ and $k_{accum}$ rather than $k_{acute,N}$, $\alpha_{accum,N}$, and $k_{accum,D}$. This is because when fitting individually, the dose per fraction, D, and initial seeding density, $N_{0}$, are fixed and known. Thus, Eqs. (2), (3), (5), and (7) are enough to characterize the data. We next discuss how the model parameters ($X_{pop}$ and $X_{ind,i}$) are updated in time.

#### 2.6.3. Updating the parameter weights to update the prediction for the next time step

**Figure 2.**
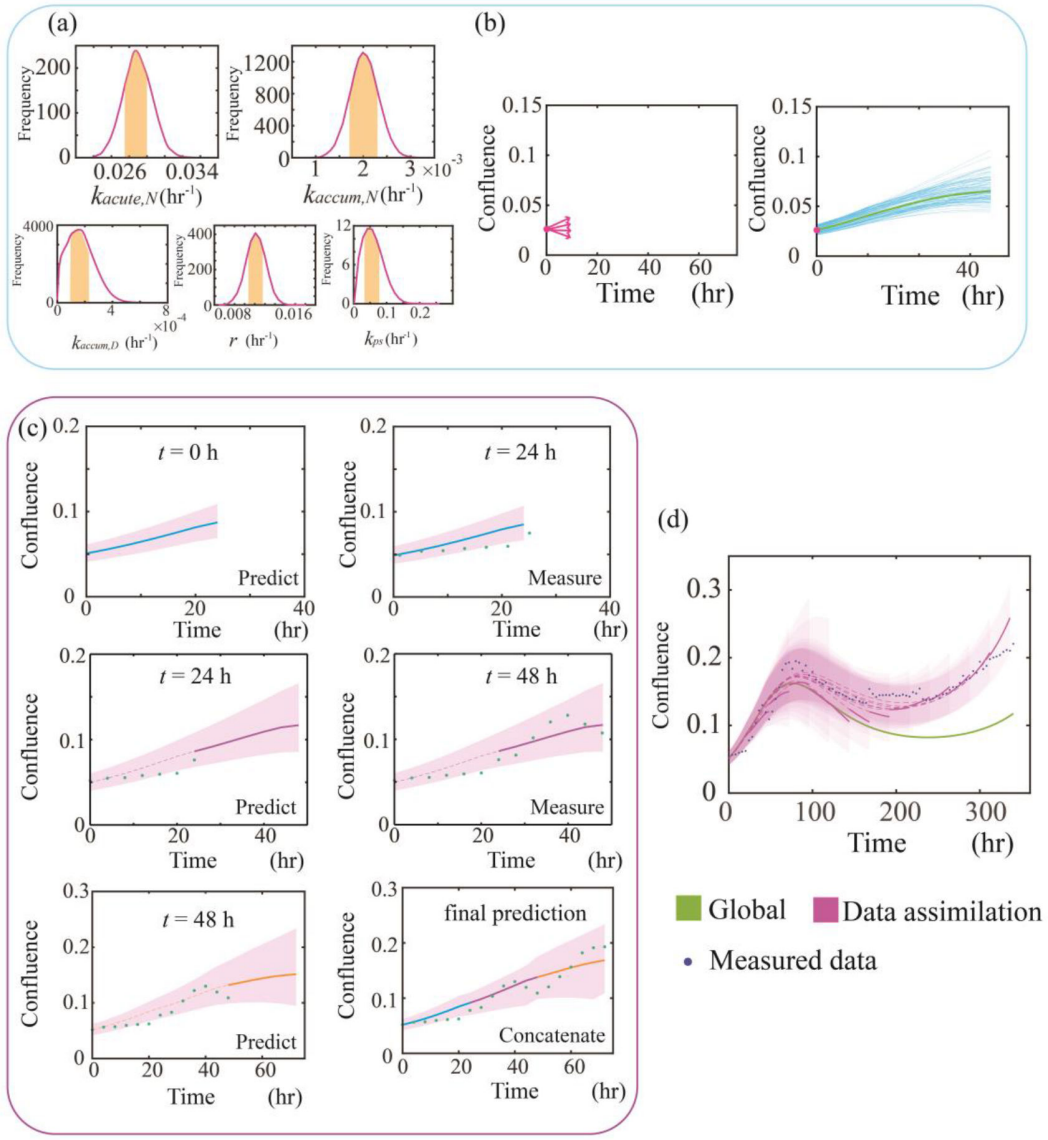
Monte-Carlo based sampling-prediction approach. In panel **(a)**, the normalized histogram for each parameter is plotted. Each parameter is sampled from the interquartile range (labeled by yellow) of the global parameter distribution $N(X_{pop},\;\sigma_{weighted})$ obtained from fitting the model to all the time courses in the training data set. The sampled value is then weighted with $X_{ind,i}$ *via* Eq. (10).. In panel **(b)**, we run the model forward 5,000 times starting from time point 0 with the weighted parameters, yielding 5,000 predictions (blue curves). The median value of these 5,000 curves (green) is our prediction, while the range of these curves forms the prediction interval. The confluence on the *y*-axis indicates the area covered by cells divided by the total area in the cell culturing well (a normalized value between 0 and 1). Panel **(c)** illustrates the prediction of the first 72 hours. At t=0, we only measure the initial condition of this new sample and then predict the first 24 hours (blue). Next, at t=24, we adjust the parameters based on new measurements of this sample and then predict from 24 to 48 h (red). The same steps are repeated for the prediction between 48 and 72 h (yellow). By concatenating prediction at different intervals (i.e., blue, red and yellow), we obtain our final prediction. In panel **(d)** we run the model forward starting from time point 0 up to (i+1)T, where i is the data assimilation step and T is the time interval between predictions (i.e., T=24 hrs in this study). We then concatenate the prediction (solid red curve) every 24 hours. Compared to global prediction (labeled in green), the data assimilation prediction captures the tumor cell regrowth observed at the tail for this specific sample.

To update the model parameters based on the global parameters $X_{pop}$ and individual parameters $X_{ind,i}$, we implement a Monte-Carlo based sampling approach as shown in Figure 2. In Figure 2a, we randomly sample from the interquartile range (25^th^ quantile to 75^th^ quantile) of the global parameters’ normal distribution $N(X_{pop},\;\sigma_{weighted})$ as the prior. $\sigma_{weighted}$ is the weighted standard deviation that decreases as time advances as shown in Eq. (9):

(9) $$\sigma_{weighted}=\frac{\sigma_{pop}}{1+\omega },$$

where ω represents our confidence on the individual parameters, $X_{ind,i}$. As time advances, we accumulate an increasing number of observations, and thus have more confidence in the sample-specific $X_{ind,i}$ compared to population-based $X_{pop}$. The updated parameters $X_{weighted}$ are then obtained by shifting the sampled values (from the global parameter distribution) towards $X_{ind,i}$, as shown in Eq. (10):

(10) $$X_{weighted}=(1-\omega )\cdot X_{pop}^{*}+\omega \cdot X_{ind,i},$$

where $X_{pop}^{*}$ represents an evaluation of $X_{pop}$ (i.e., $k_{acute,N}$, $\alpha_{accum,N}$, $k_{accum,D}$, r, and $k_{ps}$) at the sample’s radiation dose and confluency. That is, we evaluate Eqs. (4) and (6) for each individual sample to obtain $k_{acute}$ and $k_{accum}$. This is required for the following reason. When we fit the global parameters $k_{acute,N}$, $\alpha_{accum,N}$, and $k_{accum,D}$, they are independent of both dose and confluence. However, when fitting for an individual time course, this is not necessary as the dose and initial cell number is already fixed and known. Thus, fitting for $k_{acute}$ and $k_{accum}$ is sufficient for individual curves. This creates a problem that $X_{pop}$ and $X_{ind,i}$ cannot be directly weighted via Eq. (10), as they contain different parameters. Thus, $X_{pop}^{*}$ is the conversion (i.e., from $k_{acute}$ to $k_{acute,N}$) for these parameters to be weighted. After weighting the parameters, we then make predictions by running the model (i.e., Eqs. (2), (3), (5), and (7)) forward using $X_{weighted}$ from time 0 to time (i+1)T, yielding the prediction of cell confluence from t=iT to t=(i+1)T.

The weights (i.e., the level of confidence) on $X_{ind,i}$, increase linearly in time as given by Eq. (11):

(11) $$\omega =min\left(\frac{iT}{T_{total}},1\right),$$

where $T_{total}$ is the total time of our training set. (Note that when i=0 and ω=0, the weighted parameters equal the global parameters $X_{pop}$.) Eqs. (9) - (11) ensure that the model is dominated by global parameters at the earliest time points before shifting towards the individual parameters as more measurements become available.

The Monte-Carlo based sampling-predicting approach just described is repeated 5,000 times for each time step, yielding 5,000 running forward curves as shown in Figure 2b. The upper and lower bound of all the curves define our prediction intervals, and the median of these 5,000 curves yields the actual prediction. Our final prediction of the entire time course consists of the concatenation of each prediction from iT to (i+1)T every T hours. A step-by-step example of the prediction of the first 72 hours is illustrated in Figure 2c. A full example comparing the prediction with and without data assimilation is presented in Figure 2d.

#### 2.6.4. Alternative cost function for model calibration

The cost function in Eq. (8) sums the prediction error at each time point equally (i.e., the error at time point 0 contributes to the cost function the same as the error at current time point iT). However, the goal is to predict the cell confluence from iT to (i+1)T every T hours as accurately as possible. As the early time point data may not be as important as the data from more recent time points, we update Eq. (8) to reflect this observation:

(12) $$cost_{rev}=\sum_{j=1}^{\begin{array}{c} curve\\ number \end{array}}\sum_{t=0}^{iT}\left({\left(N(t)-model(X_{ind,i},t)\right)}^{2}\cdot min(t,T_{total})\right),$$

where $cost_{rev}$ denotes the “revised” cost function.

#### 2.6.5. Weighting the individual parameters

The weights on $X_{ind,i}$ in Eq. (11) capture how much confidence we have in the individual parameters versus global parameters. Initially, we have very few observations of the sample under investigation so that we have minimal confidence in the sample-specific parameters. As we have more observations, we increase our trust in the $X_{ind,i}$; thus, the weight increases linearly over time as shown in Eq. (11). We now also investigate a quadratic weighting as shown in Eq. (13):

(13) $$\omega_{rev}=-0.6\cdot {\left(\text{min}\left(\frac{iT}{T_{total}},1\right)\right)}^{2}+1.2\cdot \left(\text{min}\left(\frac{iT}{T_{total}},1\right)\right)+0.4,$$

where the coefficients (i.e., −0.6, 1.2, and 0.4, rounded to the nearest tenth) are determined by satisfying $\omega_{rev}(i=1)>0.5$ (so that the weight on the individual parameters is always greater than the population parameters), $\omega_{rev}(1)=1$, and $\max(\omega_{rev})=1$. (Note that, in this analysis, we take T=24 hrs and $T_{total}=330$ hrs.) This formulation (i.e., Eq. (13)) ensures the weights on individual parameters are more than 0.5 from the beginning of the time course and enables the model to generate more individualized predictions at early time points, as the quadratic function increases faster than the linear function. A comparison between the two weight functions is illustrated in supplementary material Figure S2.

### 2.7. Model selection and validation

Amending the basic framework (sections 2.6.1 - 2.6.3) by the various weighing schemes (sections 2.6.4 and 2.6.5) will yield four models shown in Table 1. It is not obvious, *a priori*, which one of these will yield the most accurate predictions when applied to the time-resolved microscopy data. To identify the model with the highest predictive accuracy, we compare the concordance correlation coefficient (CCC) between each model’s prediction and the measurements obtained from the test group. When comparing the similarity between the measurement and prediction curves, both the Pearson correlation coefficient (PCC) and CCC are commonly used. The PCC measures the precision, while the CCC considers both the precision and the accuracy [29], and is therefore a better metric for our study. Another common option would be the mean squared error (MSE); however, in our case, the 9L cell line grows more densely (with a carrying capacity of 0.98) compared to the C6 cell line (with a carrying capacity of 0.81). Thus, using the mean squared error between the two cell lines may not provide a fair comparison without proper normalization. To test if one model yields significantly move accurate predictions than the others, we perform the one-way ANOVA test *via* MATLAB function ‘anova1’. We select the best model (i.e., the model with highest average CCC within the testing group) and verify the prediction accuracy *via* the validation group, which is data never “seen” by the predictive framework during the data assimilation and model selection phases.

## 3. Results

### 3.1. Parameter distribution shifts over time

**Figure 3.**
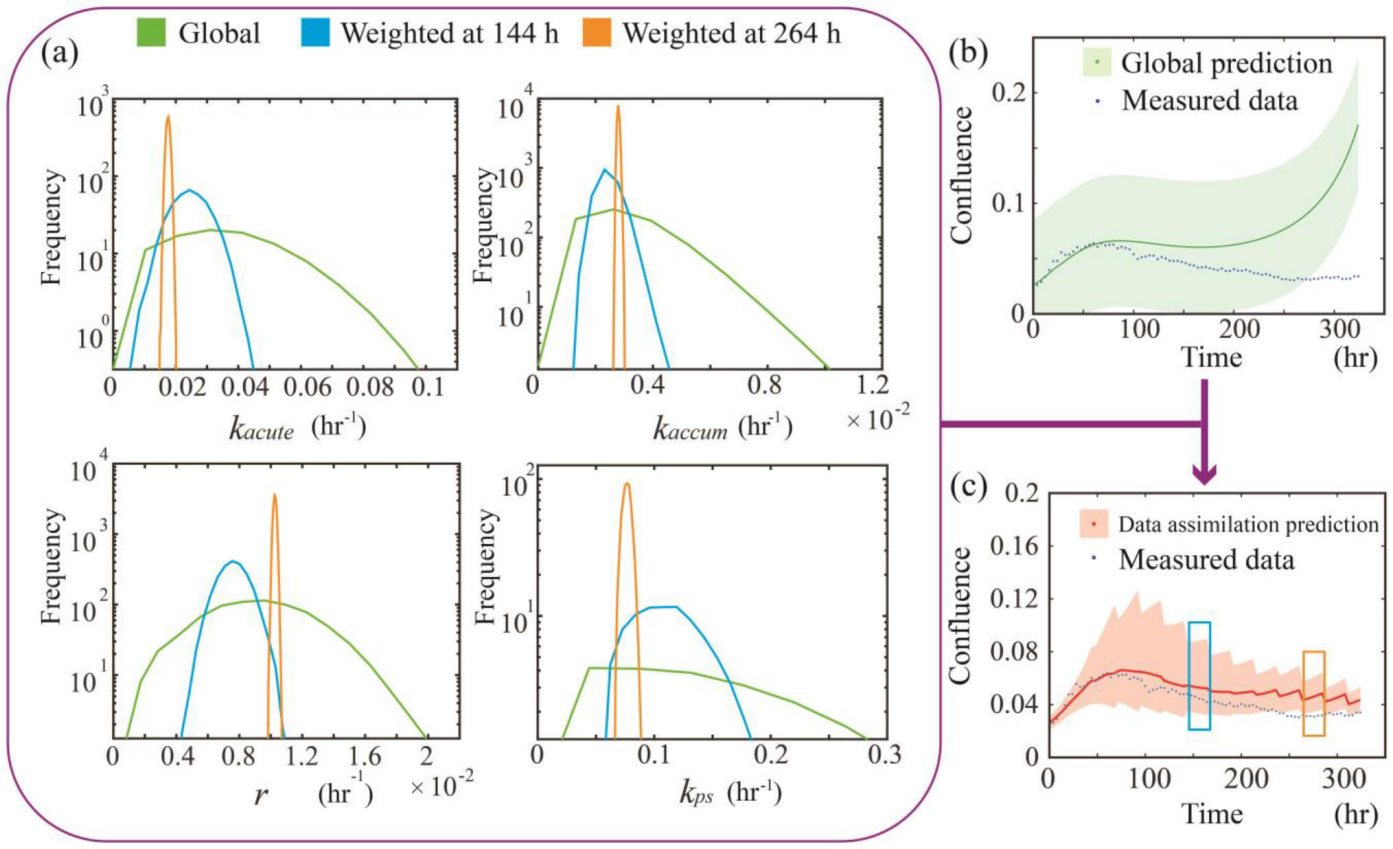
Parameter distributions and the corresponding prediction. In panel **(a)**, the weighted parameter distributions at time points 0 h, 144 h, and 264 h are labeled in green, blue, and yellow, respectively. (Note that the *y*-axis of the histogram is plotted on a log scale.) These histograms are acquired by sampling the global distribution 10,000 times, weighted according to Eq. (10), normalized and plotted (global parameters are directly plotted without weighting). Note how the distribution becomes narrower and more sample-specific as time advances. Note that due to different scales for the distribution (as all distributions are normalized to have the same area under the curve), the vertical axes are cut-off for presentation purposes. All normal distributions are truncated at zero due to their biological definitions (e.g., a negative death rate is meaningless). The global prediction in panel **(b**), which is acquired by running the model forward with global parameters (i.e., the green distribution in panel (a)), fails to capture the experimentally observed cell death (green). The data assimilation prediction (red) in panel **(c)** attempts to “correct” the wrong trend in (b), resulting in the “zig-zag” shape. The prediction during 144 – 168 h (blue box), and 264 – 288 h (yellow box) are performed using the blue and yellow parameter distribution in (a).

Figure 3a illustrates the parameter distribution of $X_{weighted}$ at 0, 144, and 264 hours. The weighted parameters start with a distribution of $X_{pop}$ (labeled in green). At 144h, $X_{pop}$ is now weighted by the individual parameters $X_{ind,6}$ and thus shifts from the global (green) to the weighted distribution (blue), which is narrower and thus more sample-specific. The distribution at 264 hours is labeled in yellow. When we only perform the Scenario 1 prediction as in panel (b), it typically reflects only the cell response of the training group and doesn’t capture the structural details of the new sample. For example, panel (b) shows how the population averaged parameter set leads to a prediction which misses the cell death occurring towards the end of the experimentally-measured time course. Conversely, Panel (c) shows a more accurate prediction obtained by the data assimilation framework (i.e., Scenario 2). Note how in the Scenario 2 case, the parameters gradually shift from the population-based distribution to the sample-specific distribution as more measurements become available. This is reflected in the CCCs for the two predictions: −0.14 for the prediction in panel (b) and 0.83 for the prediction in panel (c).

### 3.2. Model selection

The average CCC between each model’s prediction and the experimental data from the test group are listed in Table 1. In this table, model 1 is Scenario 1 in which the prediction is made using only the test case’s initial conditions and the population averaged model parameters, $X_{pop}$. Models 2-5 are the four variations of the predictive framework described in section 2.7 in which the different models are defined by how the weights and cost functions are determined. For both cell lines, the framework achieves the highest CCC when using the cost function described by Eq. (12) with the quadratic weights described by Eq. (13). Compared to the Scenario 1 predictions which achieved a CCC value (mean ± std) of 0.853 ± 0.177, model 5 achieved a significantly larger CCC of 0.950 ± 0.049 for 9L cell line (p = 0.002). Similarly, for the C6 line, the Scenario 1 predictions achieved a CCC of 0.745 ± 0.213, while model 5 achieved a significantly larger CCC of 0.931 ± 0.048 (p = 8.9e-5). Consequently, model 5 is chosen and we now seek to validate it with the data from the validation group.

### 3.3. Model validation

**Figure 4.**
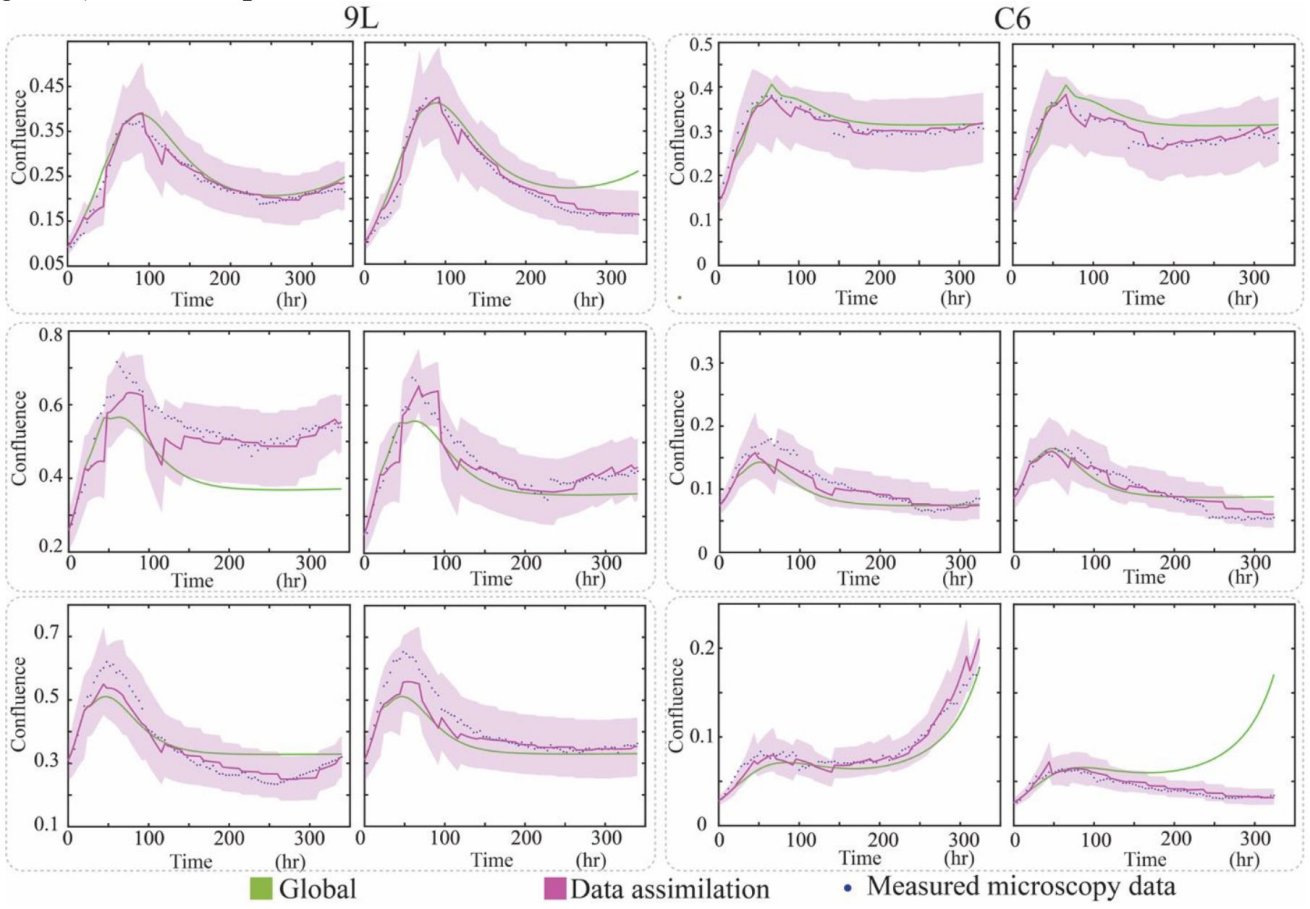
Comparison between global and data assimilation prediction. Each pair within the same dotted box presents two different samples with the same initial conditions (i.e., the same radiation schedule as well as initial seeding density), but different observed responses at later time points. While the global prediction (green) does predict the data well for some samples, it fails to be able to characterize the range of heterogeneous responses, as it fully relies on the training group curves. Conversely, the data assimilation approach (red), which individualizes the prediction on a sample-specific basis, is able to faithfully capture the range of responses.

Data in the validation group (20% of the data) is “unseen” by the modeling framework during the training and selection processes. Using the data from the validation group (36 9L samples, 25 C6 samples), the Scenario 1 prediction achieved an average CCC value (mean ± std) of 0.845 ± 0.185 for 9L cell line, and 0.726 ± 0.195 for C6 cell line. Using model 5 from Scenario 2, we achieved a significantly larger CCC value of 0.953 ± 0.052 (p = 4.8e-6) for the 9L cell line, and 0.901 ± 0.061 (p = 2.6e-10) for C6 cell line, respectively. Figure 4 illustrates the prediction results by comparing selected samples with heterogeneous response. To view all samples from validation group, please see Figures S3 and S4 from the supplementary material. Observe that the 9L validation group (0.953 ± 0.052) performs better compared to its testing group (0.950 ± 0.049). We note that this difference is very slight, not significant, and is actually due to outliers and the small sample size used in our testing set (details are provided in Supplementary Material S5).

## 4. Discussion

We have proposed and validated a data assimilation framework to individualize the prediction of heterogenous response of 9L and C6 cell lines under fractionated radiation treatment. Classical radiation models are based on the linear quadratic (LQ) model [30], which describes the surviving fraction given a specific dose and does not explicitly account for the surviving fraction as a function of time. While the LQ model has been shown to be appropriate (and practical) to predict and quantify endpoint measurements (i.e., a single data point at the end of the study) [31], it is not designed to characterize highly time-resolved longitudinal data acquired (for example) throughout fractionated treatment. As radiation-induced cell death is now well-understood to involve a combination of events at multiple time scales [32] including acute apoptosis, slow mitotic catastrophe [26], and the conversion to the senescent cells [27], it is imperative to construct mathematical models that account for these phenomena. This understanding, combined with advances in quantitative imaging techniques, makes longitudinal measurements and predictions possible.

Multiple biology-based mathematical models have been developed to characterize and predict the growth and response of tumors to treatment [33,34]. For example, on the cell scale, Yang *et al.* [35] linked the growth of cancer cells to glucose availability and the bystander effect using a set of ordinary differential equations and time-resolved microscopy data. Johnson *et al*. [36] developed a framework linked a machine learning model with a biology-based model employing single cell transcriptomic data with time-resolved microscopy data to predict the response of breast cancer cells to chemotherapy. At the tissue scale, Hormuth *et al*. [12] has incorporated the angiogenesis into a coupled set of reaction-diffusion equations parameterized by quantitative MRI data and applied in a murine model of glioma. However, the parameters in these models, once calibrated, were fixed and not updated when additional data became available during the experiment. Given this linking of biology-based models and quantitative, longitudinal data, it is now possible to begin to apply the methods of data assimilation to predict tumor growth and response dynamics. Although central to weather forecasting [37], data assimilation has rarely been applied in oncology. One such application was implemented by Zahid *et al.* [19], where the carrying capacity for a new patient after radiation treatment was personalized based on previous data. To build upon this study, we sought to incorporate multiple cell death pathways (i.e., apoptosis, mitotic catastrophe, senescence) into a biology-based model designed to accept time-resolved microscopy data, and employed data assimilation techniques to maximize predictive accuracy.

The framework presented in this study is general and not limited to the model summarized by Eqs. (2) - (7). To apply this framework to other biology-based models, one can simply: 1) obtain population-based parameters by fitting a training group globally to the model (section 2.5), 2) when new data becomes available in a testing set, obtain the individual parameters by fitting the most up-to-date set of observations in the testing set (section 2.6.2), 3) properly weighting the population based and individual model parameters (section 2.6.3), and 4) run the model forward with weighted parameters *via* a Monte-Carlo based data assimilation approach (sections 2.6.3- 2.6.5). The design of the cost function and weights depend on how much confidence we have in early measurement time points versus the more recent time points. Currently, we multiply the residual sum of squares with a linear function of time in the revised cost function Eq. (12). However, using an exponential weight of time would be a reasonable choice if the system changes quickly over time, as it would more rapidly decrease the importance of the early timepoints compared to a linear weighting. There are other methods for updating predictions as more data become available. In addition to our approach based on Monte Carlo, two other common techniques are the sequential Monte Carlo (SMC) and the Kalman filter. Sequential Monte Carlo methods incorporate the time during the sampling process (e.g., sequential importance-weighted sampling) [38]. The Kalman filter (with origins in weather forecasting [39]) seeks to account for errors with unknown origin into the modeling approach. These are both excellent ways to incorporate data assimilation into predictive models of tumor growth and response to treatment.

In general, the data assimilation approach provides a substantial improvement in describing the experimentally measured time courses when compared to the global model. It is true, though, that in the early portion of the experiment (e.g., the first 100 hours as in Figure 4), the global prediction can be more accurate than the data assimilation prediction; this is not unexpected because at the early time points we have very few observations, and the predictive accuracy can be quite modest when including just the first few observations. In particular, the first few observations tend not to display much heterogeneity across different samples so that the global prediction can do rather well. Thus, during the early time points, data assimilation does not provide much additional information. However, as we obtain and include more observations, we gain more confidence in the individual parameters so the data assimilation prediction always captures more details and outperforms the global prediction.

As the application of data assimilation to predicting tumor response dynamics is quite new, there are several opportunities for future improvement of the approach outlined in this study. First, the data assimilation framework refines predictions based only on the microscopic cell confluence data and does not account for either the phenotypic or genotypic changes for each sample. While not incorporating genomic data does make our framework flexible as it has minimal data requirements, many studies [40] have linked several genetic biomarkers to the radiation sensitivity. Thus, incorporating these biomarkers into an appropriate modeling system is likely to further improve the sample-specific predictions. In particular, determining how biomarkers affect the early death rate $k_{acute}$ and late death rate $k_{accum}$ is a natural extension of the current modeling system. Second, the model fitting and prediction scheme always uses data starting from time point 0. This increases the computational cost as more data becomes available in time. An alternative, more efficient, option is to perform data assimilation and prediction based only on a few, most recent, time points. However, we believe this is not currently practical to implement without directly measuring the temporal dynamics of the senescent component. As the senescent cell confluence is a component of the initial conditions of model, and we do not know the value beyond an assumed value of $N_{s}(t=0)=0$, we always have to run the model forward from t=0. Furthermore, the lack of direct measurement of the senescent fraction precludes an explicit description of the senescent conversion rate, $k_{ps}$, in terms of time, dose per fraction, and fraction number. Thus, we view incorporating the time-resolved measurements of senescent cell population [41] is central to advance this line of investigation. Third, the framework is verified only on cell data, which usually has higher spatial and temporal resolution and shorter study length compared to clinical studies. The clinical study usually has a much lower temporal resolution (e.g., once per week), spatial resolution, and signal-to-noise when compared to *in vitro* work. However, with the development combined MRI linear accelerator systems it may be feasible to acquire more frequent images during treatment. This will directly affect how often (and how well) data assimilation can be performed (e.g., once several weeks), as well as how we design the weighting and cost function.

## Conclusion

We have incorporated our biology-based fractionated radiation model into a data assimilation framework and verified this method *via* time-resolved microscopy data of 9L and C6 cell lines. Furthermore, the method proposed in this study is not limited to the current cell lines or mathematical model and should be applicable to other experimental settings. We hope this experimental-computational approach motivates the continued effort of developing practical methods for employing biology-based mathematical models to predict cancer growth and response to treatment. Moving forward, we aim to extend this data assimilation framework to our tissue-scale models in both the pre-clinical and clinical settings [12,15,42-44], thereby providing new methods to optimize cancer treatment on a patient-specific basis.

## Supplementary Material

1

## Figures and Tables

**Table 1.** Model selection (mean ± standard deviation)

	Cost function	ω	Average CCC for 9L	Average CCC for C6
**1**	No weights	Global	0.853 ± 0.177	0.745 ± 0.213
**2**	No weights	Linear	0.906 ± 0.109	0.833 ± 0.131
**3**	Multiply by time	Linear	0.911 ± 0.105	0.870 ± 0.093
**4**	No weights	Quadratic	0.943 ± 0.058	0.888 ± 0.061
**5**	Multiply by time	Quadratic	0.950 ± 0.049	0.931 ± 0.048
