# Supplementary material for: A data assimilation framework to predict the response of glioma cells to radiation
Source: Math Biosci Eng. Author manuscript; Available in PMC 2024 Jun 11. (PMC11165419; doi:10.3934/mbe.2023015)
Supplement: 1 [file NIHMS1995420-supplement-1.pdf]

Supplementary

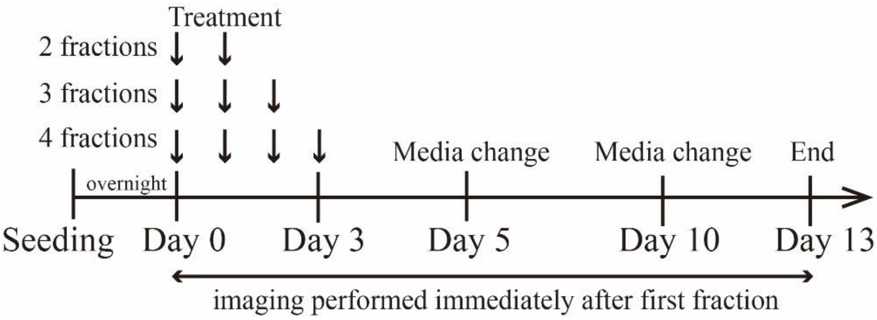

Figure S1. Radiation treatment schedule. Cells are seeded and incubated overnight before treatment. On Day 0, the cells are irradiated with either a total of 16 Gy or 20 Gy *via* different schedules. In the 16 Gy group, the cells receive either two fractions of 8 Gy, three fractions of 5.3 Gy, or four fractions of 4 Gy. In the 20 Gy group, the cells receive either two fractions of 10 Gy, three fractions of 2 Gy, or four fractions of 5 Gy. All fractions are separated by 24 hours. We refresh the cell culture media on Days 5 and 10. Microscopy images are collected continuously right after the first fraction on Day 0 until the end of Day 13.

Table S1. Model parameters.

Parameter	Units	Interpretation	Source
$\boldsymbol{k_p}$	hr^{-1}	Proliferation rate	Computed from control group, and fixed throughout experiments
$\boldsymbol{\theta}$	1	Carrying capacity	
$\boldsymbol{A}$	1	Allee effect	
$\boldsymbol{N_p}$	1	Confluence of proliferating cells	Initial cell confluence and total confluence (i.e., N_p+N_s) are measured from microscopy data.
$\boldsymbol{N_s}$	1	Confluence of senescent cells	
$\boldsymbol{N_0}$	1	Initial confluence of cells at time = 0	
$\boldsymbol{f_{DSB}}$	1	The fraction of DSBs remaining unrepaired (normalized between 0 and 1)	Measured by flow cytometry
$\boldsymbol{k_{acute}}$	1	Early death rate	Fit for individual parameters $X_{ind,i}$
$\boldsymbol{k_{accum}}$	1	Late death rate	
$\boldsymbol{k_{acute,N}}$	hr^{-1}	Death rate quantifying the contribution of initial confluence to early death	Fit for global parameters X_{pop}
$\boldsymbol{a_{accum,N}}$	1	Scale factor quantifying the contribution of initial confluence to late death	
$\boldsymbol{k_{accum,D}}$	hr^{-1}	Death rate quantifying the contribution of radiation doses to late death	
$\boldsymbol{r}$	hr^{-1}	Radiation efficacy	Fit both globally and individually
$\boldsymbol{k_{ps}}$	hr^{-1}	Conversion rate from proliferation to senescent components	

Note: A units value of “1” indicates the parameter is dimensionless.

Table S2. Pre-trained cell constants. (mean ± standard error)

**Parameter**	**9L**	**C6**
k_p	0.079 (+/- 0.011)	0.16 (+/- 0.019)
θ	0.98 (+/- 0.013)	0.81 (+/- 0.0061)
A	0.33 (+/- 0.043)	0.15 (+/- 0.0076)

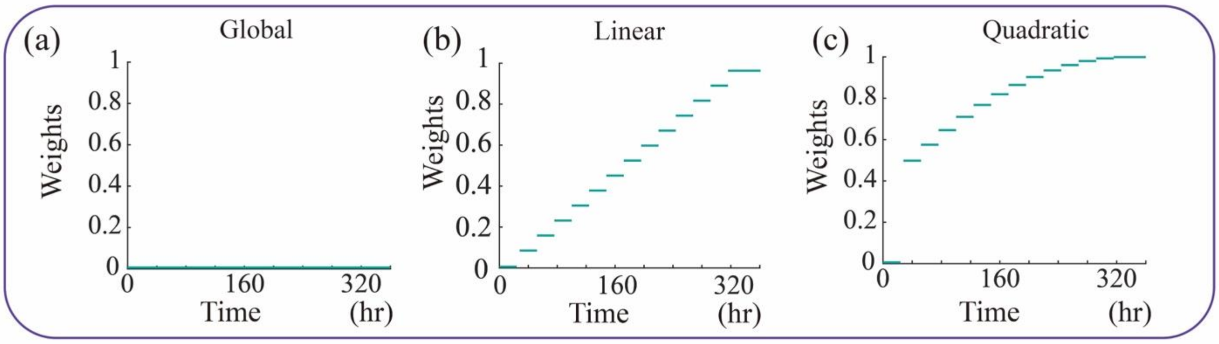

Figure S2. Illustrating different methods of weighting the individual parameters. In panel **(a)**, global prediction is performed by using only X_{pop}, which is equal to setting the weights on $X_{ind,i}$ to 0 throughout the time course. In panel **(b)**, we increase the weights linearly as in Eq. (11). In panel **(c)**, we increase the weights quadratically *via* Eq. (13). Compared to the linear weighting, the quadratic weighting scheme emphasizes the sample-specific measurements earlier in the time course.

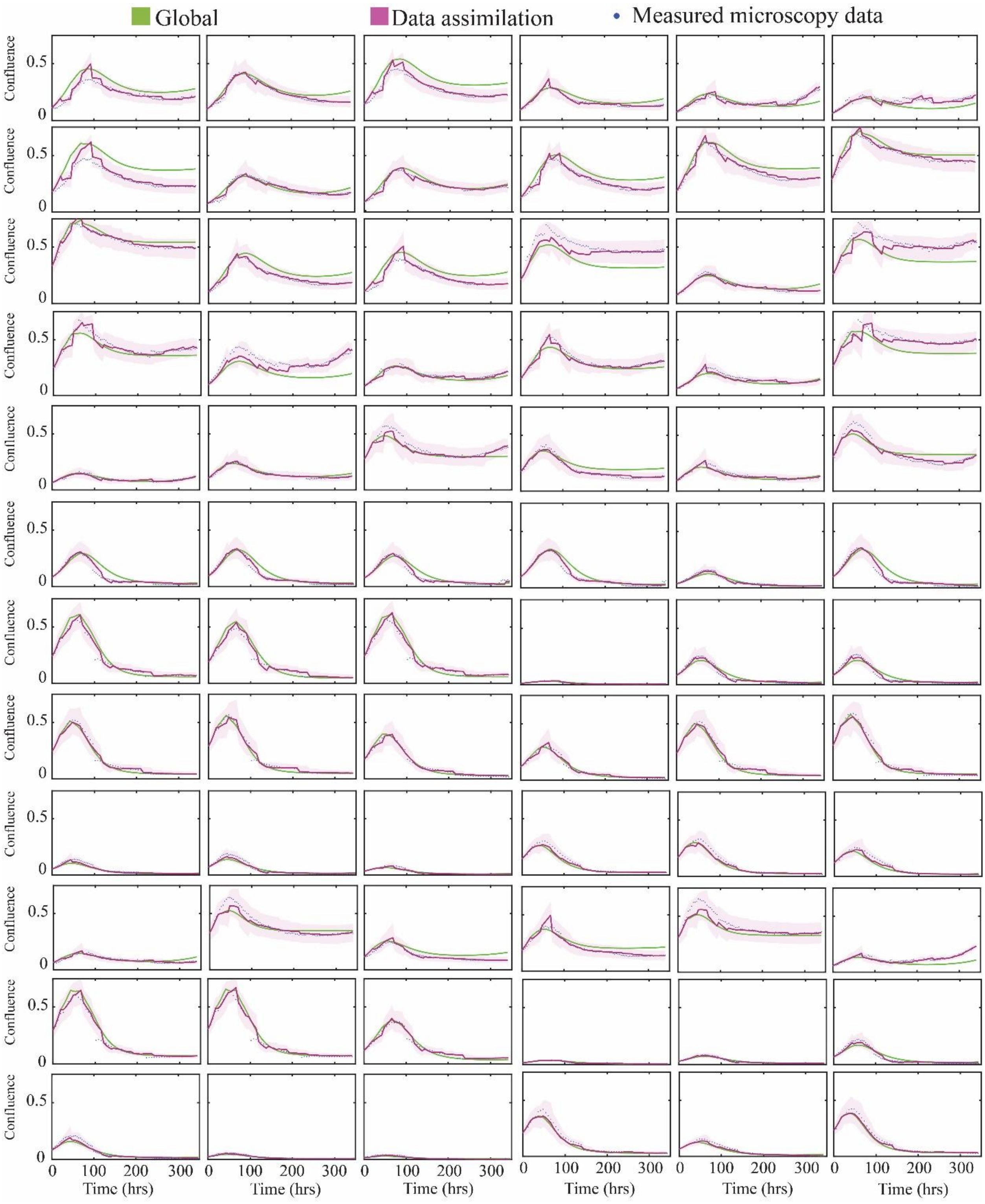

Figure S3. The data assimilation prediction versus global prediction for all 9L samples from the validation group. See caption to Figure 6 for an explanation of the results.

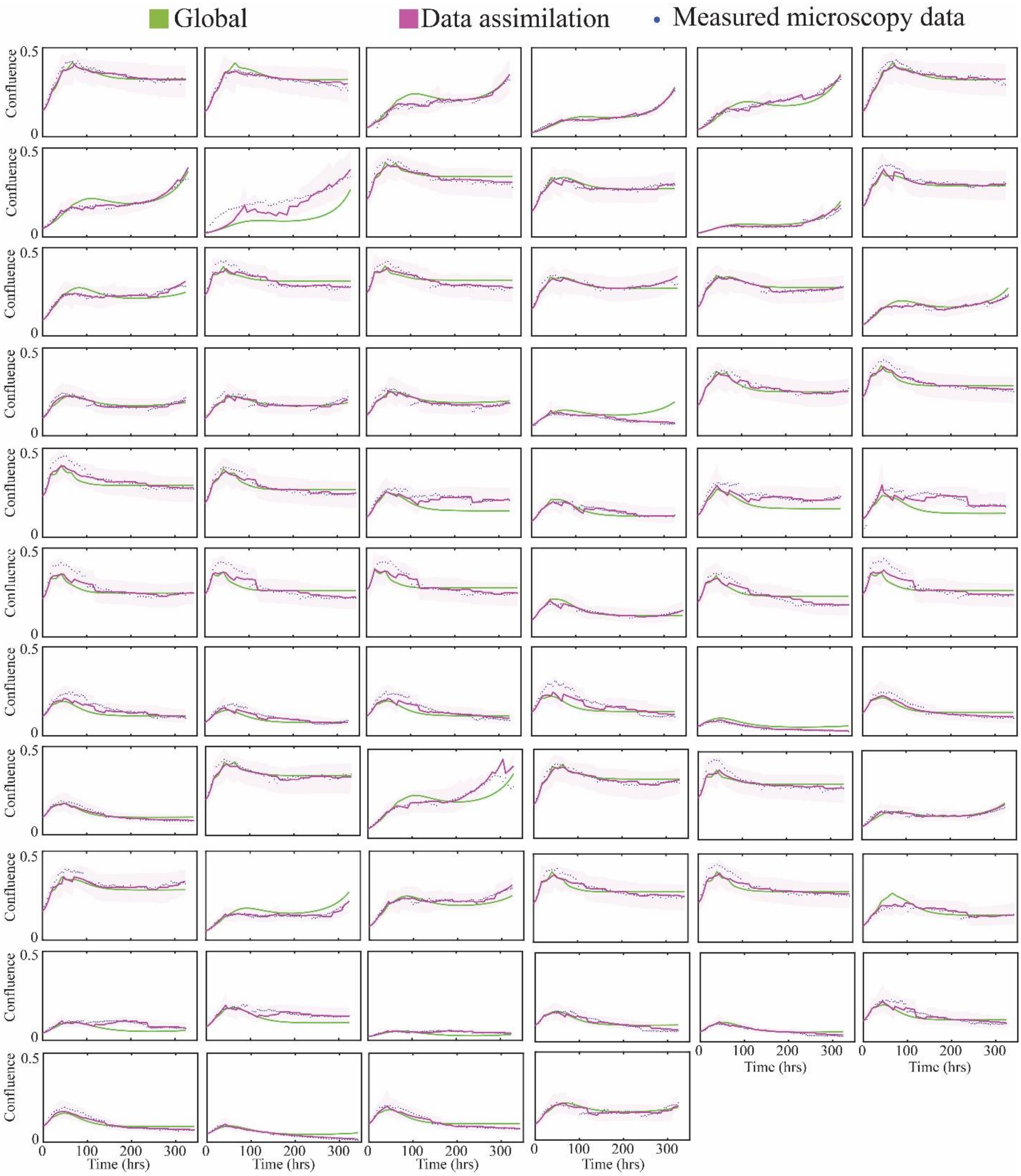

Figure S4. The data assimilation prediction versus global prediction for all C6 samples from the validation group. See caption to Figure 6 for an explanation of the results.

S5. 9L validation group versus testing group accuracy.

To understand why the accuracy of 9L validation group (0.953 ± 0.052) is better compared to its testing group (0.950 ± 0.049), which is not common in learning models, we checked the accuracy of 9L validation group and testing group for each treatment conditions, and acquired the following numbers (mean ± standard deviation):

a) four fractions of 4 Gy – testing (0.961 ± 0.027), validation (0.960 ± 0.029)
b) three fractions of 5.3 Gy – testing (0.878 ± 0.050), validation (0.875 ± 0.092)
c) two fractions of 8 Gy – testing (0.916 ± 0.041), validation (0.942 ± 0.036)
d) four fractions of 5 Gy – testing (0.984 ± 0.004), validation (0.984 ± 0.002)
e) three fractions of 6.7 Gy – testing (0.986 ± 0.005), validation (0.986 ± 0.005)
f) two fractions of 10 Gy -- testing (0.977 ± 0.013), validation (0.974 ± 0.010).

This confirms that the slight difference only comes from the two fractions of 8 Gy group of the 9L cell lines. We then checked the original data and there were no curious differences found. However, we do notice the following: because the testing group only includes 36 9L samples in total as mentioned in the Methods section (and only 6 samples are from two fractions of 8 Gy group), a ‘bad prediction’ curve may greatly affect this value. In fact, there are two such curves in these six samples (with CCCs of only 0.857 and 0.891, respectively). By removing these two ‘bad examples’, the average of the remaining four curves achieves 0.947 ± 0.027. We thus believe this slight difference is due to the two outliers and small sample size used in our testing set.
